# Pathology and Symptoms of Constipation

**Published:** 1903-12

**Authors:** Mark W. Peyser

**Affiliations:** Richmond, Virginia; Secretary Richmond (Va.) Academy of Medicine and Surgery; Examining Physician for the National Hospital for Consumptives of Denver, Colorado; Assistant Physician to the Home For the Aged and Infirm, etc.


### ATLANTA
### Journal-Record of Medicine.

**Successor to Atlanta Medical and Surgical Journal, Established 1855, and Southern Medical Record, Established 1870.**

VOL. V. DECEMBER, 1903. No. 9.

BERNARD WOLFF, M.D., EDITOR, M. B. HUTCHINS, M.D., BUSINESS MANAGER,

Nos. 319–20 Prudential. Published Monthly. 1007-1008 Century Bldg.

### *ORIGINAL COMMUNICATIONS.*

### PATHOLOGY AND SYMPTOMS OF CONSTIPATION.*

BY MARK W. PEYSER, M.D., RICHMOND, VIRGINIA.

Secretary Richmond (Va.) Academy of Medicine and Surgery; Examining Physician for the National Hospital for Consumptives of Denver, Colorado; Assistant Physician to the Home For the Aged and Infirm, etc.

Though constipation is often a symptom of other conditions, as intestinal and abdominal tumors, chronic diseases, etc., it undoubtedly exists as an idiopathic affection, and as such it will be dealt with here.

It is an habitual retention of feces in the alimentary canal; or it may be characterized by difficult or insufficient emptying of the intestines. Constipation, or costiveness, is met with in two forms, general or peristaltic, and rectal. The second form is caused, in the majority of cases, by neglect of the habit of periodic relief, the impairment of the evacuant function of the rectum being a primary

* Read before the Richmond Academy of Medicine and Surgery, July 14, 1903.

feature, and that portion of the bowel becoming a mere receptacle like the bowel above (Fox.—*Gaillard's Medical Journal*, May 1898).

The frequency with which evacuations occur in the healthy person, varies with the individual. One movement daily is the rule, but in some it may be two or even three without detriment, while in others one evacuation every two or three days, or even a week, seems not to impair the health. One case is reported in which the patient, "since his thirtieth year has been in the habit of passing stools once in six months or so" (Sajous's Analytical Cyclopedia, vol. II. p. 309).

There are no constant lesions in functional constipation; but if it endures, it may cause changes which, themselves, may later become additional causes, namely, intestinal dilatation and hypertrophy.

Colonic distension, more or less, caused by gaseous accumulation, is a common accompanyment of constipation but rarely a primary pathologic condition producing it, unless preceded by either intestinal paralysis or some form of obstruction. Pouches containing mucus and fecal matter, may form as a result of paralysis of muscular coat, and are found at the sigmoid flexure. Dilatation may begin at a distance of one or two inches from the anus (which seems spasmodically contracted), and occupy more or less of the remainder, sometimes the whole large intestine; in which latter case the chief distension is observed in the rectum, sigmoid flexure and cocum.

Hypertrophy of the muscular coat, which almost invariably occurs, is general; but most marked in the sigmoid flexure and upper part of the rectum.

Ulceration and perforation of the dilated intestine may cause fatal peritonitis. When ulceration occurs, it is perhaps partly due to yielding of the mucous membrane from overdistension, partly to the constant irritation produced by the fecal mass. Perforation may ensue while the constipation is unrelieved, from the continuance of the ulceration or from laceration; or following evacuation, from progress of the ulceration (Reynolds.—A System of Medicine).

A considerable amount of chronic irritation and subacute inflammation of the cocum, colon and surrounding cellular tissues exists in every case of fecal impaction due to habitual obstruction. When this becomes acute, as it not unfrequently does, typhlitis and peri-typhlitis are present. Peristalsis is reflexly arrested as a result of the subacute inflammation, and if a purgative be given in this condition, induction of peristalsis will only result in an incomplete evacuation, and the intestine become more torpid than before.

Other results of habitual constipation are hematuria, ulcerative colitis, rectal abcess, fistula, anal fissures, prolapsus ani, hemorrhoids, hernia, vasico-uterine tumors, prostatic hypertrophy, passive hyperemia of the pelvic viscera, cerebral hemorrhage, and in old persons mainly, as the result of colonic dilatation with saculation, enteroliths.

The symptoms of habitual constipation may be direct or local, or reflex or general. Usually, when a person whose bowels are accustomed to move daily, habitually passes two or three days without an evacuation, he experiences dyspeptic symptoms, flatulence, sometimes nausea, a sense of fullness of the rectum, etc. These symptoms, which will be more fully detailed, are also experienced by the habitually constipated, but having become habitual cease to be observed, or at all events, become more tolerable.

Colonic distension, when present, produces more or less pain which is nearly always referred to the chest. There may be interference with the functions of the duodenum, and consequently, dyspepsia, from pressure upon it of the distended transverse colon. Irritation of the bladder, and of the genito-urinary tract, neuralgic pains in the ovaries, testicles, groins, loins and lower extremities sometimes result from the pressure of large fecal masses in the descending colon and cecum.

Diarrhea is often a misleading symptom in habitual constipation. The presence of scybala which have been formed in the sacculi of the colon, may excite constant desire for evacuation, and yet they are passed only after violent efforts. In this condition, patients think they suffer with diarrhea. In those who are habitually constipated, the rectum, by reason of loss of natural sensitiveness, becomes impacted with dry, hard fecal masses of great size. Here, catarrh may be set up, and the passage of mucus excreted as a result, and sometimes accompanied by fluid fecal matter, may closely simulate diarrhea. Rarely, membranous casts of the intestine are voided from time to time. They may be several inches, or even feet, in length (Fagge.—Practice of Medicine). Osler says that the membrane is due to a derangement of the functions of the mucous glands, the nature of which is unknown; but Anders believes that membranous colitis, or entritis, which he has found to be invariably associated with a decidedly constipated habit, is a secretory neurosis, and that the catarrhal process may develop as a secondary event. The passage of the membrane is accompanied by tenesmus and severe colicky pains.

The true state of affairs may also be overlooked in what is sometimes called "cumulative constipation." In this condition the rectum, owing to the hurried and inattentive performance of defecation, is not completely emptied, resulting in a feeling of fullness and a sense of incomplete relief (Anders).

General symptoms are due, in the majority of cases, to a degree of autointoxication caused by the formation of septic materials and their absorption. There are malais, irritability, headache, vertigo, a feeling of languor, mental inactivity with insomnia, or the patient awakes unrefreshed from an unbroken sleep. The breath is foul; the tongue, which is coated and often indented by the teeth, is coated. The urine is scant and high colored. The perspiration has an offensive odor and the skin is shrivelled, pasty and sallow, or it may be, jaundiced from pressure on the biliary duct. Eruptions, such as psoriasis, eczema, prurigo, erythema, urticaria and acne, often occur. The face flushes frequently, and under the eyes are deep purplish rings. There may be frequent attacks of cardiac palpitation which cause anxiety on the part of the patient. Shortness of breath is, at times, a prominent symptom.

Those who are habitually constipated are subject to attacks of vertigo and temporary loss of consciousness (Loomis—Practice of Medicine). In marked cases attacks of nausea and vomiting, with diarrhea, may ensue; fever may also be present, and even typhoid fever be simulated (Meigs).

It is sometimes a question whether hypochondria and neurasthenia cause constipation or are caused by constipation. The nervous affection is often probably the primary disease which is followed by constipation; while in other cases the habitual constipation leads secondarily to the nervous depression. The two conditions usually form a vicious circle, since each of them is able to keep up and to increase the other (Strumpell.—Practice of Medicine).

Enlargement and pouching of the lower third of the rectum is a result of chronic constipation, which may not only counterfeit, but produce uterine trouble. It is found very often in virgins, and gives the pain in the back, discomfort on standing or walking (more particularly on standing), and the sensation of dragging and fullness as if the parts would fall. This is due to distension and varicosity of the vaginal and uterine veins caused by the formation of a protocele which presses forward the vagina. Intense pain is caused by efforts at defecation which force the vagina and rectum downward to the pubis and perineum. These efforts, instead of relieving the patient, force the uterus downward, and, through traction on the vagina, produce prolapse or retroversion. It is easily understood that since the cause is primarily the constipation, correction of the displacement will not relieve the patient.

*303 Twelfth Street, north.*

